# Boron Neutron Capture Therapy: A Technology-Driven Renaissance

**DOI:** 10.3390/cancers18030498

**Published:** 2026-02-03

**Authors:** Dandan Zheng, Guang Han, Olga Dona Maria Lemus, Alexander Podgorsak, Matthew Webster, Fiona Li, Yuwei Zhou, Hyunuk Jung, Jihyung Yoon

**Affiliations:** 1 Department of Radiation Oncology, University of Rochester, Rochester, NY 14627, USA; alexander_podgorsak@urmc.rochester.edu (A.P.); matt_webster@urmc.rochester.edu (M.W.); fenglifiona_li@urmc.rochester.edu (F.L.); yuwei_zhou@urmc.rochester.edu (Y.Z.); hyunuk_jung@urmc.rochester.edu (H.J.); jihyung_yoon@urmc.rochester.edu (J.Y.); 2 Department of Radiation Oncology, Hubei Cancer Hospital, Huazhong University of Science and Technology, Wuhan 430079, China; hg7913@hotmail.com; 3 Department of Radiation Oncology, University of Miami, Coral Gables, FL 33146, USA; oxd407@med.miami.edu

**Keywords:** BNCT, boron, neutron, precision medicine, theranostics, personalized therapy

## Abstract

Boron neutron capture therapy (BNCT) is experiencing a global resurgence driven by advances in boron pharmacology, accelerator-based neutron sources, and molecular imaging-guided theranostics. BNCT produces high linear energy transfer particles with micrometer-range energy deposition, enabling cell-selective irradiation confined to boron-enriched tumor cells in a geometrically targeted region by the neutron beam. This mechanism offers the potential for exceptionally high therapeutic ratios, provided two core requirements are met: sufficient differential tumor uptake of ^10^B and a neutron beam with appropriate energy and penetration. After early clinical attempts in the mid-20th century were hindered by inadequate boron agents and reactor-based neutron beams, recent technological breakthroughs have made BNCT clinically viable. The development of hospital-compatible accelerator neutron sources, next-generation boron delivery systems (such as receptor-targeted compounds and nanoparticles), advanced theranostic approaches (such as ^18^F-BPA positron emission tomography and boron-sensitive magnetic resonance imaging), and AI-driven biodistribution modeling now support personalized treatment planning and patient selection. These innovations have catalyzed modern clinical implementation, exemplified by Japan’s regulatory approval of BNCT for recurrent head and neck cancer and the rapid expansion of clinical programs across Asia, Europe, and South America. Building on these foundations, BNCT has transitioned from a predominantly academic experimental modality into an increasingly commercialized and industrially supported therapeutic platform. The emergence of dedicated BNCT companies, international collaborations between accelerator manufacturers and hospitals, and pharmaceutical development pipelines for next-generation boron carriers has accelerated clinical translation. Moreover, BNCT now occupies a unique position among radiation modalities due to its hybrid nature, namely combining the biological targeting of radiopharmaceutical therapy with the external-beam controllability of radiotherapy, thereby offering new therapeutic opportunities where competitive approaches fall short. Emerging evidence suggests therapeutic promise in glioblastoma, recurrent head and neck cancers, melanoma, meningioma, lung cancer, sarcomas, and other difficult-to-treat malignancies. Looking ahead, continued innovation in compact neutron source engineering, boron nanocarriers, multimodal theranostics, microdosimetry-guided treatment planning, and combination strategies with systemic therapies such as immunotherapy will be essential for optimizing outcomes. Together, these converging developments position BNCT as a biologically targeted and potentially transformative modality in the era of precision oncology.

## Simple Summary

Boron neutron capture therapy (BNCT) is a specialized form of radiotherapy that aims to selectively destroy cancer cells while sparing surrounding normal tissues. It works by delivering a boron-containing drug that accumulates preferentially in tumor cells, followed by neutron irradiation that triggers highly localized, cell-killing radiation only where boron is present. Earlier attempts at BNCT were limited by inadequate boron drugs and bulky reactor-based neutron sources, but recent advances have renewed interest in this approach. Modern accelerator-based neutron systems, improved boron delivery agents, and imaging techniques such as PET and MRI now allow clinicians to better select patients and personalize treatment. BNCT has already been approved in Japan for recurrent head and neck cancer and is being explored worldwide for other difficult-to-treat tumors, such as brain tumors and melanoma. With continued technological and clinical development, BNCT has the potential to become an important precision radiotherapy option for cancers with limited conventional treatment choices.

## 1. Introduction: Fundamental Principles and Theoretical Strengths Underlying Its Renewed Clinical Interest

The basic reaction principle of Boron Neutron Capture Therapy (BNCT) is summarized in Equation (1) [1,2]. When a stable ^10^B nucleus absorbs a low-energy (thermal) neutron, it undergoes a nuclear reaction to yield a ^7^Li nucleus and an alpha particle (^4^He), along with the release of kinetic energy:

(1) B510+n01→Li37+He24

**Figure 1:**
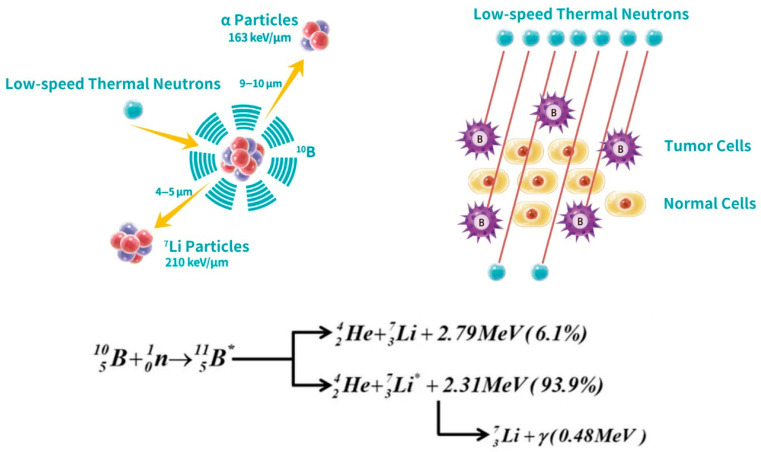
Schematic of the boron neutron capture therapy (BNCT) mechanism. The (**left**) panel depicts the nuclear reaction where a ^10^B nucleus captures a thermal neutron, producing high-LET particles, an α particle (^4^He) and a ^7^Li nucleus, with a short penetration range (4–10 μm). The (**right**) panel illustrates the therapeutic selectivity: thermal irradiation specifically targets tumor cells pre-loaded with ^10^B, sparing adjacent normal cells that lack significant boron accumulation. The underlying reaction pathways of ^10^B nucleus absorbing a neutron, including their respective energies and branching ratios, are also shown.

**Figure 2:**
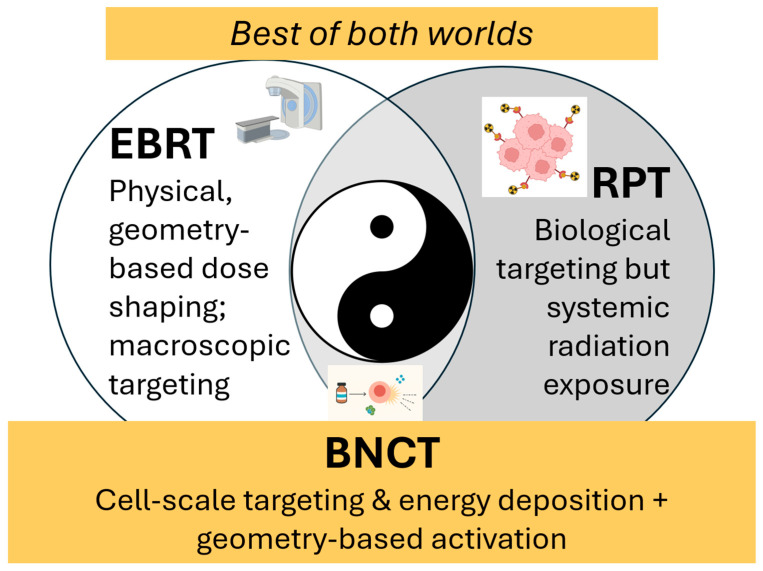
BNCT is uniquely positioned to achieve a high therapeutic ratio. Because of its dual selectivity of biological targeting at the cellular level combined with external-beam spatial control, BNCT occupies a unique position among radiation modalities. Like an optimal balance of yin and yang, BNCT’s hybrid nature creates unique therapeutic opportunities in scenarios where EBRT is limited by cumulative dose constraints or infiltrative tumor spread, and where RPT may be restricted by systemic distribution, off-target uptake, or insufficient local dose escalation.

Both the lithium and helium products have high linear energy transfer (LET) and deposit their energy within approximately 4–10 µm, a distance comparable to or smaller than a single mammalian cell diameter. This means that the cytotoxic effect of BNCT occurs only where boron is present, enabling a biologically and spatially targeted therapy as depicted in Figure 1. On their own, neither the boron-containing compounds nor the neutron beam is particularly harmful at therapeutic dose levels. Only when the two are combined within boron-enriched tumor cells does the therapy generate lethal, highly localized radiobiological damage. As illustrated in Figure 2, this spatial confinement of high-LET energy deposition and neutron beam-directed activation makes BNCT inherently suited to achieving a high therapeutic ratio. In contrast to external-beam radiotherapy (EBRT), where dose gradients are governed by macroscopic beam geometry and tissue heterogeneity, BNCT delivers cytotoxic radiation directly at the cellular and subcellular scale, with a dose fall-off sharper than that achieved even with heavy-ion therapy [3]. Moreover, compared to radiopharmaceutical therapy (RPT), BNCT offers distinct advantages deriving from its hybrid nature. RPT depends entirely on the biodistribution of radioactive agents, which continuously emit radiation until decay, contributing to systemic radiation exposure. In contrast, BNCT enables temporal and spatial control over the activation step: boron carriers are non-radioactive, and high-LET radiation is generated only during neutron irradiation. Non-targeted tissues that uptake boron are spared from high-LET damage, reducing collateral toxicity. Thus, BNCT combines the biological precision of molecular targeting with the procedural controllability of external beam activation. This mechanism illustrates a dual advantage not shared by EBRT or RPT alone, and underlies BNCT’s unique appeal as a precision radiation modality capable of delivering cell-selective dose while preserving normal tissue integrity.

Meanwhile, while conceptually ideal as a modality with very high therapeutic ratios, the success of BNCT fundamentally depends on two components:

1. Achieving sufficient differential accumulation of ^10^B in tumor cells relative to surrounding normal tissues.
2. Delivering a neutron beam with appropriate energy and penetration for the anatomical depth of the tumor.

These requirements are technically and biologically demanding, and realizing them in widespread clinical practice requires sustained research and innovation in boron pharmacology, neutron source engineering, and treatment planning. The BNCT concept, initially proposed in 1936, saw its first clinical trials in the 1950s [1,2,4,5]. However, early BNCT efforts were hampered by technological limitations: namely, suboptimal boron delivery and the lack of neutron sources strong and practical enough for clinical use. As a result, BNCT remained a niche experimental modality for decades. Today, we are witnessing a technology-driven renaissance of BNCT. Advances in both neutron source engineering and boron carrier chemistry have revived global interest and propelled BNCT from academic research into hospital clinics. In 2020, Japan became the first country to approve an accelerator-based BNCT system (NeuCure™ by Sumitomo Heavy Industries, Tokyo, Japan) together with a boron drug (Steboronine^®^, a borofalan drug, Osaka, Japan) and dedicated treatment planning software for unresectable recurrent head-and-neck cancers, with coverage under its national health insurance [6,7,8]. This milestone symbolizes how improved technology has transformed BNCT into a viable clinical therapy and frames the modern “renaissance” discussed in this article.

## 2. Historical Attempts and Lessons Learned

BNCT is not a new concept but has a history of close to a century. However, when it came out, it was conceptually ahead of its time. The first human BNCT treatment occurred in 1951 at Brookhaven National Laboratory [4,5]. Researchers initially targeted high-grade gliomas, which remain among the most challenging cancers to treat. However, the early clinical trials faced critical limitations. Besides the common challenge of the blood–brain barrier (BBB), the boron agents available at the time also lacked tumor specificity, leading to high boron concentrations in blood and normal tissues, particularly in brain vasculature. Meanwhile, the thermal neutrons generated from research reactors had limited penetration depth, causing suboptimal dosing of deeper tumors and excessive irradiation of the normal scalp and meninges. These challenges led to severe toxicities and eventual halting of BNCT trials in the United States.

However, research continued in Japan, Finland, China, Argentina, and parts of Europe [9,10,11,12,13,14,15,16,17,18,19]. Japanese and other investigators made significant progress in refining neutron beam shaping assemblies, patient treatment planning, and boron pharmacokinetics. Together, these efforts continued to propel BNCT development and translation and eventually led to its recent resurgence.

## 3. The BNCT Renaissance: Current Global Landscape

For many decades, the field progressed slowly, largely due to technological and pharmacologic limitations. Only in the last decade has BNCT re-emerged as a viable and increasingly realistic radiotherapeutic modality, largely thanks to ongoing active research and commercialization in Japan and other Asian and European countries [9,10,11,12].

One distinguishing feature of BNCT’s modern revival is the strong participation of industry and the shift from purely academic research to commercial development. Historically, BNCT trials were conducted almost exclusively in academic or government settings, often constrained by limited resources and access to reactors. Today, companies and private investors have entered the field, accelerating progress.

Japan has seen the most progress and success in this aspect with its BNCT commercial rollout. The collaboration between Sumitomo Heavy Industries with the NeuCure™ (Tokyo, Japan) neutron accelerator and Stella Pharma with the boron agent Steboronine™ (Osaka, Japan) led to the first fully integrated, regulatory-approved BNCT platform, comprising neutron source, boron drug, and planning software [20,21]. China has followed this model by importing the complete Japanese package into a pilot medical zone in Hainan as an interim step toward domestic BNCT capacity [22]. Stella Pharma and TAE Life Sciences have also announced agreements to co-develop and commercialize BPA and other boron agents in the U.S. and Europe, indicating growing global business interest [23].

There have also been increasing start-ups and joint ventures specialized in BNCT technology across the globe. Neutron Therapeutics (U.S./EU) provides the Hyperion™/nuBeam™ accelerators (Danvers, MA, USA) and has partnered with Helsinki University Hospital in Finland and a Japanese medical group, creating a multi-national BNCT network [17]. TAE Life Sciences, a subsidiary of TAE Technologies, has developed the AlphaBeam™ BNCT beamline (Irvine, CA, USA) and is collaborating with the University of Wisconsin–Madison to establish the first accelerator-based BNCT clinic in North America, while also participating in Asian programs such as Xiamen’s BNCT center in China [24]. In China, government and private sectors have jointly funded BNCT centers. Neuboron and the China National Nuclear Corporation built the NeuPex system (Nanjing, China), and other companies are collaborating with the China Institute of Atomic Energy on cyclotron-BNCT units [22]. Over a dozen enterprises, research institutes, and universities in China are involved in BNCT device development and are moving toward device licensing [22].

**Figure 3:**
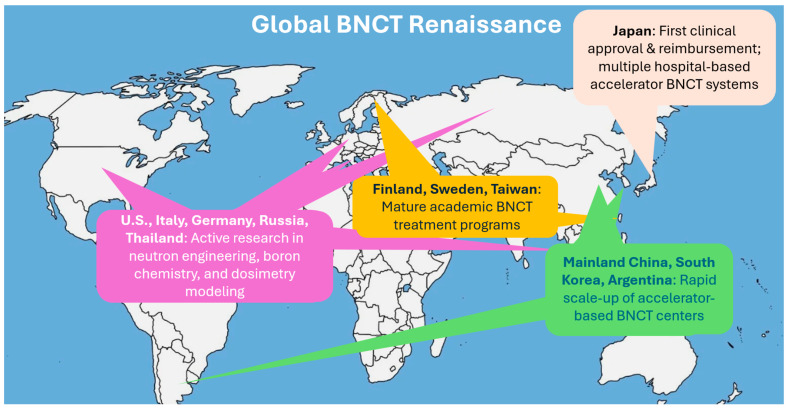
The current global renaissance of BNCT.

Along with the development progress, there have also been increasing global clinical trials. With industry support, BNCT’s clinical evidence base is rapidly expanding. Japan has treated hundreds of patients, primarily with gliomas and head-and-neck cancers, across reactor and accelerator BNCT trials. The 2020 regulatory approval in Japan for the reimbursable clinical use of BNCT for locally advanced or recurrent head and neck cancers was a major milestone. It signaled not only clinical maturity but also governmental confidence in BNCT’s therapeutic value and safety profile. In 2021, the first accelerator-based BNCT trial for recurrent head-and-neck cancer reported ~70% response rates with acceptable toxicity [25]. Finland has reported its transition from a reactor program to accelerator-based BNCT, leveraging decades of experience to streamline new trials [8,26]. In 2023, China launched or planned multiple clinical studies for advanced nasopharyngeal carcinoma and other cancers using BNCT [22]. Meanwhile, Finland, Sweden, Taiwan, Argentina, South Korea, and several European research centers have also launched or expanded BNCT clinical programs, accelerating data accumulation and expanding indications under investigation [10,13,14,15,16,17,18,19,27]. Figure 3 depicts this current global renaissance of BNCT. Even the United States, which had been largely inactive in BNCT for many years, is now preparing clinical BNCT centers. This represents a structural change that BNCT is moving towards routine clinical environments with robust backing from both industry and academic medicine.

This renewed interest is driven by several converging advances which will be detailed in the following section: (1) the development of accelerator-based neutron sources that make BNCT clinically deployable outside nuclear reactor facilities, (2) improved boron delivery agents with enhanced tumor selectivity and higher intracellular accumulation, (3) the broader rise in theranostic medicine, in which targeted therapy and molecular imaging are integrated for personalized treatment, and (4) AI-driven biodistribution modeling and personalized BNCT planning [13,14,28,29].

## 4. Technological Advances Driving Modern BNCT

Limitations such as nonspecific boron distribution and bulky, expensive reactor-based neutron sources with limited-penetration constrained early BNCT. But recent innovations in selective boron carriers and hospital-installable accelerator-based neutron sources transformed these once-prohibitive limitations into solvable engineering and pharmacologic challenges. These technological advances driving the modern BNCT renaissance are discussed in more detail in this section.

### 4.1. Advances in Neutron Sources: From Reactor-Based to Accelerator-Based

Until the early 2000s, nuclear research reactors were essentially the only neutron sources capable of supporting BNCT, severely limiting its clinical dissemination. Although reactor-based beams offered high neutron intensities, they were expensive to operate, operationally inflexible, and rarely accessible for routine clinical scheduling. In many countries, reactor construction near or within medical centers is restricted or prohibited, consistent with the International Atomic Energy Agency (IAEA) guidance that emphasized epithermal neutron flux requirements without addressing hospital deployment challenges [30].

Over the past two decades, concerted physics and engineering efforts worldwide have led to the development of compact accelerator-based neutron sources (ABNSs) specially built for BNCT, with wide commercialization and clinical adoption previously described in Section 3. This development represented a major turning point in the BNCT field. These systems employ light-charged particle accelerators, including cyclotrons, linear accelerators (linacs), and electrostatic accelerators. They produce high-intensity proton or deuteron beams that strike targets such as beryllium (Be) or lithium (Li) to generate neutrons via (p,n) nuclear reactions [10,31,32].

The development of ABNS devices suitable for clinical BNCT necessitates not only accelerated beams up to ~30 MeV range with average currents of several milliamperes, but also robust target systems capable of withstanding substantial thermal loads and preventing material degradation. In practice, Be targets are favored above ~10 MeV protons for higher neutron yield, while Li targets are effective at lower energies due to their favorable cross sections, but require careful engineering to manage activation products and heat dissipation [33].

Table 1 summarizes a comparison between different sources of neutron generation for BNCT. Earlier nuclear reactor neutron beams were logistically complex, expensive, and often politically infeasible. Unlike them, modern compact ABNSs are designed to be installed within hospitals or cancer centers and to produce controllable neutron fields that can be shaped into epithermal spectra using tailored beam-shaping assemblies (BSAs) of moderators and filters. The neutron energies typically in the 0.5 eV to 10 keV range were recommended by IAEA for deep-seated tumor irradiation, allowing neutrons to penetrate deeply before thermalizing in the tumor, maximizing the boron-10 capture reaction and minimizing healthy tissue damage [30]. The ability to tailor the spectrum via BSAs is critical: accelerator-produced neutrons initially span a broad energy range and must be moderated and filtered to achieve clinical quality beams with minimized fast neutron and gamma contamination.

**Cyclotron-based systems.** Cyclotron-based systems have been among the most clinically mature platforms. A prominent example is the Sumitomo Heavy Industries HM-30 cyclotron, which accelerates protons up to ~30 MeV; when coupled with a Be target and optimized BSA, it has achieved epithermal neutron fluxes on the order of 10^9^ n/cm^2^/sec with typical currents ~1 mA, enabling installation in hospital environments such as the Kansai BNCT Medical Center and Southern Tohoku Hospital [35,36]. This class of system received Japanese regulatory approval in 2020 and has been reimbursed under national health insurance for locally advanced or recurrent head and neck cancers [7,26].

**Linac-based neutron sources.** Linac-based systems represent another robust technology pathway for hospital-based BNCT. Radio-frequency quadrupole (RFQ) front ends combined with downstream accelerating structures such as drift-tube linacs (DTLs) have been developed to accelerate protons in the ~2.5–8 MeV range with high average currents (mA-class to tens of mA), enabling neutron production via p-Be or p-Li reactions suitable for clinical BNCT beamlines [37,38]. These architectures support modular integration of accelerator, target, and BSAs, and are being engineered for operational flexibility, maintainability, and hospital installation. Ongoing projects in Japan and elsewhere are focused on improving linac stability, current delivery, and beam-shaping performance to generate clinically useful epithermal neutron beams with a reduced facility footprint and enhanced suitability for routine clinical operation.

**Electrostatic accelerators.** Electrostatic accelerators provide yet another modality for BNCT neutron generation [39]. These systems typically use tandem or single-ended electrostatic designs to accelerate lower-energy protons (e.g., ~2–3 MeV) onto lithium targets. While the neutron yield per unit current is lower relative to higher-energy cyclotrons or linacs, the simpler shielding requirements, lower operating power, and compact footprint make electrostatic sources attractive for certain clinical installations. Multiple electrostatic BNCT projects are under development, and some have entered clinical service or are approaching clinical trials [40].

Together, these ABNS systems offer improved beam shaping, better intensity control, and increased cost-efficiency. Commercial partnership in Japan has led to clinical system deployment and ongoing expansion into Europe and Southeast Asia.

### 4.2. Advances in Boron Delivery Agents: From Empirical Compounds to Targeted Therapeutics

Parallel to neutron source innovation, progress in BNCT has been critically driven by the evolution of boron delivery chemistry. Because BNCT efficacy depends on the microscopic co-localization of boron-10 atoms and thermalized neutrons, boron carriers must achieve not only favorable tumor-to-normal tissue ratios but also appropriate cellular, subcellular, and microregional distribution. This requirement for microdistribution control has been emphasized since early mechanistic analyses of BNCT radiobiology [13,14]. Historically, therapeutic boron agents can be grouped into three broad generations, reflecting a progression from empirical compounds toward biologically informed and molecularly engineered systems (Table 2).

**First-generation compounds.** First-generation boron compounds, including boric acid and simple borates, distributed largely by passive diffusion and lacked intrinsic tumor selectivity. Early clinical and preclinical studies demonstrated rapid systemic clearance and short tumor retention times on the order of only 2–4 h, resulting in very low tumor-to-blood (TBR) and tumor-to-normal tissue ratios (TNR) [12,14]. Substantial boron accumulation occurred in normal tissues, particularly in vascular compartments, severely limiting the achievable therapeutic window. These pharmacokinetic limitations were directly implicated in the disappointing outcomes of early U.S. reactor-based BNCT trials for gliomas [13]. While these early compounds established proof-of-principle for neutron capture therapy, they also underscored the necessity of biologically selective and kinetically favorable boron delivery.

**Second-generation agents (clinical foundations with defined transport biology).** Second-generation carriers, primarily boronophenylalanine (BPA) and sodium borocaptate (BSH), enabled essentially all modern clinical BNCT experience and remain the clinical reference standards. Importantly, they rely on distinct biological delivery mechanisms. BPA is an amino-acid analog whose tumor uptake is strongly influenced by L-type amino acid transporter 1 (LAT1) expression, enabling intracellular accumulation and penetration across an intact BBB as demonstrated in both in vitro and in vivo transport studies [41,42]. In contrast, BSH is a boron cluster compound that generally does not cross an intact BBB, and tumor delivery in brain malignancies depends largely on BBB disruption and extracellular extravasation [13,48].

Clinically, second-generation agents represented a major advance, achieving substantially improved tumor-to-normal tissue ratios compared with first-generation compounds and enabling meaningful therapeutic responses. Large clinical series from Japan and Finland established BPA- and BSH-based BNCT as viable salvage options for gliomas, melanoma, and recurrent head and neck cancers [17,26,49]. However, extensive clinical experience has also revealed important limitations. These include pronounced inter- and intratumoral heterogeneity of uptake, variability in tumor-to-normal ratios between patients, and imperfect microdistribution within complex tumor architectures demonstrated using ^18^F-BPA positron emission tomography (PET) and tissue assays [41,44]. Notably, a substantial subset of screened patients failed to achieve commonly used TNR thresholds (e.g., ~2.5), precluding safe BNCT delivery, reinforcing intrinsic biological constraints of second-generation agents and the need for improved boron carriers and robust pre-treatment assessment strategies [41].

**Third-generation agents (targeted and high-payload strategies).** Third-generation boron agents emphasize molecular targeting, payload amplification, and microdistribution control. Rather than relying primarily on endogenous transport pathways or vascular permeability, these approaches aim to engineer boron delivery systems that actively exploit tumor-associated receptors, microenvironmental features, and intracellular trafficking mechanisms [50,51].

Representative strategies include next-generation amino-acid-based boron drugs with improved aqueous solubility and intracellular retention such as boronotyrosine analogs with superior uptake and washout kinetics compared with BPA [45,52]; ligand- or receptor-targeted boron conjugates based on peptides, proteins, or antibodies [29]; and boron-rich nanoformulations designed to deliver high boron payloads per carrier. Polymeric and hybrid nanoparticles, typically in the 50–200 nm size range, have been developed to leverage enhanced permeability and retention effects, while surface functionalization enables active targeting to tumor-associated biomarkers [44,53]. Additional platforms incorporate stimuli-responsive designs, such as pH-sensitive or enzyme-responsive systems, to promote preferential boron release within the tumor microenvironment [29]. These multifunctional nanocarriers have been developed to co-deliver boron and chemotherapeutic agents, enabling spatially coordinated high-LET irradiation and localized chemotherapy within the tumor microenvironment [48].

Rather than focusing solely on average tumor boron concentration, these third-generation approaches increasingly target improvement of spatial homogeneity, intracellular localization, and persistence within heterogeneous tumor tissue, including regions adjacent to vascular and BBB interfaces [29,44]. While many of these platforms demonstrate promising preclinical performance, they also introduce translational challenges, including synthetic complexity, pharmacokinetic control, off-target accumulation, toxicity evaluation, and regulatory maturation. Importantly, heterogeneity of uptake remains a field-wide challenge that third-generation agents continue to address.

**Positioning within modern BNCT.** Collectively, the evolution of boron delivery chemistry reflects a shift from empiricism toward biologically rational and engineering-enabled therapeutics. In addition, early in vitro and in vivo studies have demonstrated synergistic or additive antitumor effects when BNCT is combined with DNA-damaging or radiosensitizing chemotherapeutic agents, supporting the feasibility of chemo-BNCT combination strategies [44,53]. Modern BNCT increasingly emphasizes control over cellular and microregional boron localization, providing a biochemical foundation for the current BNCT renaissance.

However, the impact of these advances cannot be fully realized without accurate in vivo measurement and computational modeling of boron biodistribution. The complementary developments in BNCT theranostics and AI-assisted biodistribution modeling are discussed in the following sections.

### 4.3. Advances in BNCT Theranostics and Imaging-Guided Personalization

Because boron concentration in tissues cannot be reliably inferred from systemic blood levels, imaging-based quantification is essential to guide BNCT. Unlike conventional radiotherapy, in which physical dose can be planned largely from anatomical information, the biologically effective dose in BNCT depends critically on the spatial, cellular, and microregional distribution of boron within tumors and normal tissues. Consequently, theranostics for BNCT integrates diagnostic imaging and targeted therapy into a unified framework, enabling patient selection, treatment feasibility assessment, and individualized dose planning [29].

**PET-based boron imaging and clinical integration.** Among available approaches, positron emission tomography using fluorine-18-labeled boronophenylalanine (^18^F-FBPA) remains the most clinically established theranostic tool. ^18^F-FBPA PET provides a noninvasive surrogate of BPA biodistribution, allowing quantitative assessment of tumor uptake, tumor-to-normal tissue ratios, and intratumoral heterogeneity prior to BNCT. The tracer was originally developed to mirror BPA transport biology and has been shown to correlate with actual boron biodistribution in tumors [54]. Clinically, ^18^F-FBPA PET has become integrated into BNCT workflows in Japan and Europe for eligibility screening, dose planning, and post-treatment assessment [35,41,55]. Importantly, PET imaging enables exclusion of patients unlikely to achieve adequate therapeutic ratios and allows identification of lesions or subregions with insufficient boron accumulation.

A commonly used feasibility benchmark is a TNR ≥ 2.5 on ^18^F-FBPA PET, which has been associated with favorable boron accumulation and has been widely adopted for patient selection [41,56,57]. Recent clinical studies further demonstrate that PET-defined heterogeneity of boron uptake significantly influences absorbed dose estimation and should be incorporated into BNCT planning strategies [44].

Beyond BPA, newer PET-compatible boron tracers have been developed to improve stability, specificity, and pharmacokinetic performance. These include fluorinated boronotyrosine derivatives and other labeled amino-acid or receptor-targeted boron compounds, which aim to better reflect tumor metabolic phenotypes and to support next-generation boron agents [35,54]. Such tracers expand the theranostic toolkit and provide a pathway toward carrier-specific imaging, rather than reliance on a single surrogate.

**Magnetic resonance imaging (MRI)- and spectroscopy-based approaches**. Complementary to PET, magnetic-resonance-based techniques have been explored to characterize boron biodistribution. ^11^B magnetic resonance spectroscopy (MRS) offers a direct, isotope-specific method to detect boron compounds in vivo, providing spatial and temporal information on boron accumulation without ionizing radiation, which has been demonstrated in both preclinical and translational BNCT studies [54,58]. A preclinical rodent study also suggested that combining ^19^F MRI and/or ^19^F MRS with ^19^F-BPA could enable high-resolution mapping of boron distribution and pharmacokinetics, supporting improved BNCT treatment planning in future clinical trials [58]. Although currently limited by sensitivity and spatial resolution, advances in high-field MRI, coil optimization, and spectroscopic acquisition methods continue to improve feasibility for mapping intratumoral boron distributions [54]. In parallel, hybrid PET/MRI systems have been explored to improve anatomical co-registration and quantitative accuracy of boron imaging [59].

**Multifunctional theranostic boron platforms.** Beyond small-molecule tracers, substantial effort has been devoted to multifunctional theranostic platforms that integrate boron delivery with intrinsic imaging capability. These include boron-rich carborane clusters, boron-containing liposomes and polymeric nanoparticles, dendrimers, and hybrid inorganic–organic systems functionalized with PET isotopes, MRI contrast elements, or optical reporters. Such systems enable multimodal imaging, high boron payload delivery, and in some designs, microenvironment-responsive release triggered by pH or enzymatic activity [29].

Preclinical investigations have demonstrated that theranostic nanocarriers can enhance tumor boron retention, improve microdistribution, and support image-guided optimization of BNCT delivery [29,44]. These multifunctional platforms aim to address persistent challenges simultaneously: increasing boron/imaging probe loading efficiency, increasing tumor boron concentration, improving intracellular localization, reducing off-target exposure, and providing real-time feedback on biodistribution and response, supporting their potential role in next-generation BNCT paradigms.

**Toward precision BNCT.** Ideal BNCT theranostic agents exhibit high tumor-to-normal tissue selectivity, sufficient achievable boron concentrations (commonly cited targets ≥ 20–30 μg ^10^B/g tumor or ~10^9^ boron atoms per cell), chemical and radiochemical stability, and minimal systemic toxicity [13,29]. When coupled with quantitative imaging, these agents transform BNCT from a fixed-protocol modality into a biologically adaptive therapy, in which patient-specific boron pharmacokinetics and spatial heterogeneity inform treatment selection, beam configuration, and dose prescription.

Collectively, BNCT theranostics integrate molecular imaging, drug delivery, and radiotherapy physics into a unified precision-oncology framework. These advances provide the essential measurement layer that links evolving boron chemistry to emerging computational and AI-assisted biodistribution modeling strategies discussed in the following section.

### 4.4. Advances in AI-Driven Biodistribution Modeling and Personalized BNCT Planning

Because BNCT dose depends on the patient-specific spatial distribution of boron at voxel and sub-voxel scales, there is growing movement away from “uniform TBR” assumptions toward image-informed, heterogeneity-aware dose modeling and data-driven personalization. A central technical challenge is that boron uptake is heterogeneous within gross disease, and PET activity “hotspots” do not necessarily map one-to-one onto the eventual high-dose regions once neutron transport, moderation, and tissue composition effects are accounted for. Recent clinical dosimetry work has therefore emphasized workflows that propagate voxelized boron surrogates from ^18^F-BPA PET into dose engines and explicitly quantify how heterogeneity alters conventional dose indices. A 27-patient study comparing conventional uniform-boron assumptions with PET-informed heterogeneous dose evaluation found that incorporating heterogeneous intratumoral boron distribution yielded significantly lower tumor dose indices than conventional evaluations (*p* < 0.01), and that spatial correspondence between metabolic tumor volumes on PET and isodose volumes was limited. These types of results support the concept that dose-based (not PET-only) criteria may be needed for patient selection [55]. For boron agent development, AI tools such as machine learning–assisted molecular docking, molecular dynamics simulations, and in silico structure–activity modeling can be used to facilitate the design of high-quality agents [60].

Methodologically, several lines of AI-assisted development are converging in this space, detailed in the following paragraphs.

**Heterogeneity correction and PET-to-dose translation.** A key modeling advance is the use of PET-derived heterogeneity maps and segmentation strategies to reduce bias introduced by assuming a single tumor boron concentration. Recent advances increasingly replace uniform tumor boron assumptions with voxel-wise, image-informed modeling frameworks. Teng et al. explicitly proposed and validated a correction strategy that propagates PET-derived voxel-level boron surrogates into BNCT dose calculations, explicitly accounting for spatial heterogeneity within gross disease rather than relying on mean tumor-to-blood ratios [44]. This work formalizes how heterogeneity modifies tumor dose estimates derived from PET-like inputs. Complementary work integrating multimodality target definition (MRI vs. ^18^F-BPA PET) employs semi-automated segmentation, multimodal image fusion, and threshold-based classification strategies (e.g., TBR/TNR-based criteria) to define biological target volumes and patient eligibility, demonstrating substantial intra- and inter-tumoral heterogeneity in boron [46]. These workflows typically employ semi-automated image segmentation, multimodal image fusion, and rule-based classification strategies, reflecting AI-adjacent image analysis rather than population-level assumptions. Together, these studies support a direction where patient selection and planning become image-quantitative and heterogeneity-aware, rather than population-assumption-based.

**Time-varying (pharmacokinetic) modeling for dose accumulation.** Beyond static uptake, boron levels change over infusion and irradiation windows. Incorporating time-varying boron concentration into dose computation is therefore increasingly recognized as necessary for more faithful patient-specific dosimetry. Chen et al. developed dose calculation approaches based on pharmacokinetic time courses of boron concentration, highlighting that time dependence can be explicitly embedded into dose estimation rather than treated as a fixed scalar [61]. Methodologically, this corresponds to temporal modeling of patient-specific boron concentration curves using regression-based and pharmacokinetic learning frameworks, providing a foundation for future AI implementations incorporating time-series or state-space models.

**Machine learning (ML) acceleration and surrogate dose prediction.** Full Monte Carlo (MC) transport remains computationally expensive for iterative optimization, uncertainty analysis, and scenario testing. Tian et al. demonstrated a supervised neural-network–based prediction method to calculate therapeutic dose for BNCT patients with glioblastoma, illustrating the feasibility of machine learning surrogate models that learn nonlinear mappings between patient-specific inputs and MC-derived dose outputs for clinical workflows [62]. Practically, such approaches are best positioned as assistive accelerators to enable faster plan iteration and sensitivity analysis, while still requiring benchmarking against MC or validated deterministic engines for safety-critical deployment.

**Radiomics-based characterization of uptake heterogeneity and image biomarker development.** Radiomics extracted from ^18^F-FBPA/^18^F-BPA imaging can quantify heterogeneity beyond mean ratios, enabling modeling of uptake patterns that may correlate with feasibility, robustness of coverage, or outcomes. A study comparing conventional and radiomic features between ^18^F-FBPA PET/CT (computed tomography) and PET/MRI in malignant brain tumor patients examined cross-modality feature behavior and proposed interchange approaches, supporting the idea that high-dimensional radiomic features including texture, intensity, and spatial heterogeneity descriptors can serve as AI-derived image biomarkers for downstream modeling and decision support [63].

Overall, the near-term “AI value proposition” in BNCT is not generic automation; it is structured personalization: (i) converting functional imaging into voxelwise boron surrogates, (ii) propagating that information through validated dose engines that respect neutron transport physics, (iii) integrating time dynamics of boron delivery, and (iv) using ML models to accelerate computation and extract stable heterogeneity biomarkers. As these components mature, AI-assisted biodistribution modeling is poised to strengthen BNCT’s precision-oncology identity by making patient selection, plan robustness evaluation, and dose prescription increasingly individualized rather than assumption-driven.

Together, the efforts described in Section 4.1, Section 4.2, Section 4.3 and Section 4.4 are making BNCT more accessible and positioning BNCT within the broader movement of precision oncology, where treatment is individually tailored based on biological imaging and monitoring.

## 5. Clinical Applications and Current Status

Modern BNCT has re-emerged as a clinical option in oncology precisely because it occupies a distinct therapeutic niche relative to other radiation modalities such as EBRT and RPT (see Table 3). Whereas EBRT delivers dose primarily based on geometric beam shaping and RPT relies on systemic biologic targeting, BNCT combines macroscopic neutron beam geometry with cellular-level boron localization, thereby potentially overcoming constraints imposed by normal tissue tolerance and heterogeneous drug uptake in challenging tumors.

Contemporary clinical interest in BNCT focuses on disease contexts characterized by prior radiation exposure, limited surgical options, intrinsic radioresistance, or diffuse microscopic invasion where conventional approaches are inadequate. Accelerator-based neutron sources have facilitated a transition from largely experimental reactor studies to prospective clinical trials and approved therapy programs, particularly in Japan, Finland, and China [16,17,26].

**Recurrent head and neck cancer (the most mature clinical indication).** Recurrent or locally advanced head and neck squamous cell carcinoma (HNSCC) represents the most mature BNCT clinical application [7,8,26,64,65,66]. Multiple phase I/II studies over the past two decades demonstrated objective response rates ranging from ~60% up to 76% in inoperable, previously irradiated HNSCC patients treated with BPA-based BNCT [8]. In Japan, the JHN002 open-label phase II trial using a cyclotron-based epithermal neutron source and borofalan showed favorable objective responses and safety profiles, contributing to the 2020 regulatory approval and national insurance reimbursement of BNCT for locally advanced or recurrent head and neck cancers [8,26]. Post-marketing surveillance further supports BNCT’s effectiveness and tolerability in this setting [7].

**Central nervous system tumors and infiltrative disease**. GBM has been one of the earliest and most intensively studied indications for BNCT [9,67]. Early reactor-based trials in the U.S., Europe, and Japan were among the first clinical explorations of neutron capture approaches for brain tumors, demonstrating feasibility and acceptable safety in heavily pretreated patients [67]. Retrospective analyses of reactor-based BNCT for recurrent GBM reported median survival times on the order of 10–20 months in small cohorts, with manageable neurotoxicity compared with historical controls. More recently, accelerator-based BNCT using cyclotron neutron sources with borofalan (SPM-011) has been investigated for recurrent malignant gliomas, reinforcing safety and preliminary efficacy in phase I/II settings [68]. Early phase I protocols are also exploring combinations of BNCT with EBRT and temozolomide as multimodality approaches in newly diagnosed GBM [69].

**Superficial and radioresistant diseases.** BNCT has also been investigated in malignancies with high phenylalanine analog uptake or superficial spread. Historical reactor-based studies of malignant melanoma demonstrated high local control rates, particularly for cutaneous disease, and this experience encouraged further exploration of BNCT in radioresistant tumors [12]. Clinical experience in rare tumors such as angiosarcoma and chondrosarcoma is more limited but includes case reports and small pilot series reporting meaningful local regression, underscoring BNCT’s potential role in ultra-refractory disease contexts where other radiotherapies are ineffective [70,71,72].

**Exploratory indications.** Early clinical reports have described BNCT applications in recurrent meningiomas, some locally advanced lung cancers adjacent to critical structures, and recurrent chest-wall breast cancer [73,74]. These indications remain in pilot or early clinical evaluation, focused on feasibility, safety, and dose optimization rather than definitive outcomes, but they illustrate how modern accelerator sources are enabling broader oncologic exploration.

Table 4 provides a summary of the key clinical applications of BNCT. Across these settings, the overarching theme is that BNCT’s biophysical and biologic selectivity may offer advantages in tumors that are infiltrative, previously irradiated, or structurally complex, particularly when supported by boron imaging and individualized planning. Ongoing clinical research, coupled with advances in neutron source technology and boron delivery agents, continues to refine BNCT’s role in multidisciplinary cancer care.

## 6. Conclusions and Future Directions

Future directions for BNCT focus on several complementary and interdependent areas of advancement. First, ongoing chemical and nanotechnological efforts aim to refine tumor-selective boron carriers to achieve higher TNR, improved intracellular localization, and more homogeneous microdistribution within heterogeneous tumors. Particular emphasis is being placed on targeted, high-payload, and stimuli-responsive systems that can better penetrate complex tumor architectures and overcome biological barriers.

Second, there is a growing need to standardize and further develop treatment-planning frameworks that explicitly incorporate microdosimetry and boron biodistribution. Unlike conventional radiotherapy, BNCT dose is governed by both neutron transport and subcellular boron localization. Future planning systems will need to integrate patient-specific imaging, pharmacokinetic modeling, and microdosimetric formalisms to more accurately represent biological dose and therapeutic ratios and to generate cumulative dosimetry with other radiation modalities for combined therapy.

Third, progress in pre-treatment and intra-treatment imaging will be essential to quantify boron distribution in vivo and support biologically informed clinical decision-making. Techniques such as ^18^F-labeled BPA PET provide critical spatial maps of boron biodistribution. These data can be used to identify patients suitable for BNCT, to estimate tumor and normal-tissue boron concentrations, obtain the optimal concentration ratio, and to inform hybrid treatment strategies. In this context, BNCT may be rationally combined with EBRT, in which photon or particle irradiation is used to supplement dose in tumor subregions demonstrating inadequate boron accumulation, as defined by functional imaging.

Fourth, accelerator developers continue to pursue more compact, reliable, and cost-effective neutron sources, with the goal of reducing facility footprint, improving beam availability, enhancing the cost-effectiveness of BNCT, and facilitating broader hospital-based deployment.

Fifth, combination strategies with systemic therapies represent an emerging frontier. Preclinical and early translational studies suggest that BNCT-induced high-LET cellular damage may synergize with immune checkpoint inhibitors, molecularly targeted agents, or radiosensitizers. Exploring BNCT within multimodality regimens may expand its role beyond local control toward integrated disease management.

Finally, coordinated multi-institutional clinical trials are critically needed to define optimal disease indications, treatment timing, patient selection criteria, and comparative effectiveness relative to established external beam and radiopharmaceutical therapies. Such efforts will be essential to move BNCT from early adoption toward evidence-based integration into contemporary radiation oncology practice.

Together, these developments position BNCT as a biologically targeted and technologically enabled radiotherapy modality whose continued evolution will determine the depth and durability of its current renaissance.

## Figures and Tables

**Table 1:** Comparison of neutron source technologies for BNCT. All neutron sources, including reactor-based and accelerator-based sources, produce fast neutrons that must be spectrally shaped using BSA to generate clinically suitable epithermal or thermal beams in accordance with IAEA recommendations. Modern ABNS, including cyclotron-based, linac-based, and electrostatic systems, enable hospital-based BNCT deployment and represent the technological foundation of the current BNCT renaissance.

Feature\Source Types	Reactor-Based [30,34]	Accelerator-Based		
		Cyclotron [26,32,35,36]	Linac [18,37,38]	Electrostatic [19,39]
Neutron production mechanism	Fission neutrons from nuclear reactor core (e.g., ^235^U fission)	High-energy proton beam (~14–30 MeV) on Be target with (p,n) reactions; high neutron yield per particle	Lower-energy, high-current proton beams (~2.5–8 MeV, mA-tens of mA) on Be or Li targets with (p,n) reactions	Low-energy proton beams (~2–3 MeV) from electrostatic accelerators on Li targets with ^7^Li(p,n)^7^Be reaction or Be targets with ^9^Be(p,n)^9^B reaction
Primary neutron spectrum (before BSA)	Broad continuous fission neutron spectrum, extending from thermal energies to ~10 MeV, with a mean energy around ~2 MeV	Continuous fast neutron spectrum from (p,n) reactions on Be, with peak energies typically several MeV and a high-energy tail dependent on proton energy (≈14–30 MeV)	Continuous fast neutron spectrum from low-energy, high-current (p,n) reactions, generally softer than cyclotron-based spectra due to lower proton energies (≈2.5–8 MeV)	Lower-energy continuous neutron spectrum (~2–3 MeV, often near reaction threshold, resulting in reduced high-energy neutron components
Achievable clinical beam spectrum (after BSA)	Thermal or epithermal spectra achievable, depending on moderator design and beam port geometry; historically flexible but constrained by fixed reactor layouts	Epithermal beams commonly achieved using large, optimized BSA; thermal beams also possible for superficial applications	Epithermal beams achievable with tailored BSA, often requiring spectral compensation for lower neutron yield	Primarily epithermal beams targeted, using compact BSA designs optimized for near-threshold neutron production
Beam size and collimation	Large, fixed beam ports defined by reactor shielding and beam channels; limited flexibility in beam diameter and shaping	Moderate-to-large beam apertures, constrained by target heat load and BSA size; typically single fixed clinical beam	Greater flexibility in beam shaping, enabled by modular accelerator–target layouts and adaptable BSA designs	Compact beam apertures, often optimized for superficial or moderately deep targets; footprint-driven collimation constraints
Beam control and operation	Continuous high-flux operation; limited on/off flexibility	High stability; controllable beam energy and intensity; rapid start/stop	High average current capability; flexible pulsed or continuous wave operation	Direct current (DC) or quasi-DC beams; fine current control; simplified operation
Facility integration	National-scale facilities; not hospital-installable	Hospital-installable; multiple clinically deployed systems	Hospital-installable; modular accelerator-target layouts	Highly compact; attractive for sites with space/power constraints
Clinical suitability	Enabled early clinical trials; now largely discontinued	Most clinically mature ABNS platform; first regulatory approvals and insurance-covered BNCT in Japan	Active development for next-generation hospital systems; strong for scalability and domestic manufacturing	Entering clinical service and trials (China, Finland; planning in US, Russia, Argentina, and Italy)
Representative strengths	Very high neutron flux; historically foundational	High neutron yield; proven hospital deployment; strong clinical track record	High current operation; modularity; engineering flexibility	Compact footprint; lower shielding and power demand; simplified infrastructure
Key limitations	Political/regulatory barriers; cost; hospital infeasibility	Target heat load; large shielding; infrastructure cost	Reliability and long-term clinical validation still evolving	Lower neutron yield per mA; demanding Li target engineering

**Table 2:** Generational progression of therapeutic boron delivery agents for BNCT. Second-generation compounds (BPA and BSH) established modern clinical BNCT through distinct biological transport mechanisms, while third-generation platforms emphasize molecular targeting, payload amplification, and control of microdistribution.

Generation	Representative Therapeutic Agents	Primary Delivery Mechanism and Defining Characteristics	Key Limitations and Unresolved Challenges
First generation [12,14]	Boric acid; simple borates	Passive diffusion; rapid systemic clearance; short tumor retention times (hours); no intrinsic tumor-targeting properties	Very low tumor-to-blood and tumor-to-normal tissue ratios; substantial normal-tissue exposure; poor cellular and microregional localization; clinically inadequate therapeutic window
Second generation (clinical foundations) [13,17,41,42,43]	BPA (boronophenylalanine); BSH (sodium borocaptate)	BPA: amino-acid analog transported predominantly via LAT1, enabling intracellular accumulation and penetration across an intact BBB. BSH: boron cluster compound with limited membrane permeability; low toxicity, suitable for hypoxic/necrotic regions in tumors; tumor delivery in CNS lesions depends largely on BBB disruption and extracellular extravasation	Marked inter- and intratumoral uptake heterogeneity; variable tumor-to-normal ratios across patients; imperfect intracellular retention and microdistribution. A clinically significant fraction of patients fail to reach minimal tumor boron levels required for safe and effective BNCT
Third generation (targeted and high-payload strategies) [29,44,45,46,47]	Next-generation amino-acid boron drugs; ligand- or receptor-targeted boron conjugates (peptides, proteins, antibodies); boron-rich nanoparticles and hybrid carriers	Engineered for active tumor targeting, increased boron payload per delivery event, and improved cellular and microregional distribution. Includes receptor-mediated uptake, nanocarriers exploiting enhanced permeability and retention, and stimuli-responsive platforms designed to release boron preferentially within the tumor microenvironment	Translational complexity (synthetic scalability, reproducibility, pharmacokinetics, toxicity, regulatory maturation); risk of off-target accumulation; requirement for rigorous in vivo validation. Uptake heterogeneity remains a central unresolved challenge

**Table 3:** Comparison of EBRT, radiopharmaceutical therapy, and BNCT.

Dimension	EBRT (Photons and Particles)	Radiopharmaceutical Therapy (RPT)	BNCT
Primary targeting basis	External beam geometry and dose shaping	Systemic biological targeting	Dual targeting: neutron beam geometry + boron localization
Scale of selectivity	Millimeter–centimeter (fields, margins)	Organ/lesion/cellular (uptake-dependent)	Cellular/subcellular (boron-dependent reaction)
Radiation quality	Mostly low LET (high LET for heavy ions)	β or α emitters (agent-dependent)	High LET α and ^7^Li particles generated in situ
Dependence on tumor biology	Limited	High	High
Normal tissue dose/toxicity	Beam path and range uncertainties	Off-target organ uptake	Neutron field plus boron in normal tissues
Planning paradigm	Anatomy-based physical dosimetry	Imaging-based pharmacokinetic dosimetry	Hybrid: neutron transport + boron biodistribution
Clinical maturity	Standard of care	Established for select cancers; rapidly expanding	Approved for recurrent H&N cancer in Japan; expanding trials
Key strengths	Wide accessibility; precise geometric control; scalability	Systemic targeting; micrometastatic disease	Cellular-level selectivity; high-LET effect; re-irradiation potential
Key limitations	Normal tissue tolerance	Systemic toxicity; uptake heterogeneity	Boron delivery dependence; specialized infrastructure
Best-fit clinical niches	Most localized solid tumors	Disseminated, element-seeking, or receptor-expressing disease	Currently locally advanced/recurrent, radioresistant, or re-irradiation-limited tumors

**Table 4:** Current key clinical applications of BNCT.

Disease Site	Clinical Rationale for BNCT	Current Status and Key Evidence
Recurrent head and neck squamous cell carcinoma [7,8,26,64,65,66]	Locoregional recurrences in previously irradiated fields; limited salvage options; proximity to critical organs (carotid artery, spinal cord, oral cavity); need for high tumoricidal dose with strict normal tissue sparing	Most clinically mature indication. Prospective Japanese trials using accelerator-based BNCT led to 2020 regulatory approval and national insurance reimbursement. Reported objective response rates ~60–80% (>90% reported for recurrent larynx cancer), with meaningful symptom relief and manageable mucosal/skin toxicity. Multiple centers now treat unresectable recurrent disease clinically.
Glioblastoma multiforme (GBM) [68,75,76]	Highly infiltrative growth beyond MRI-defined margins; limited surgical resectability; poor outcomes with photon re-irradiation; relative radioresistance and hypoxia; need for cellular-level selectivity in eloquent brain regions	Reactor-based and accelerator-based clinical trials conducted in Japan and Finland; studies demonstrated feasibility, acceptable normal-brain toxicity, and median survivals comparable or superior to historical re-irradiation series, with reported median overall survival of 19 months. Finnish experience showed a safe transition to accelerator BNCT and established PET-guided patient selection. Ongoing work focuses on optimizing boron delivery, dosimetry, and combination strategies.
Malignant melanoma (cutaneous and mucosal) [12,77,78,79]	High amino-acid and phenylalanine analog uptake; superficial or multifocal lesions; relative resistance to low-LET radiotherapy; suitability for localized neutron fields	Early Japanese reactor-based trials demonstrated high response rates in cutaneous melanoma. More recent studies and case series suggest durable local control in selected patients with overall control rates of 70%-high 80% (complete response or no-recurrence partial response). Clinical use remains investigational, often in refractory or multifocal disease.
Recurrent meningioma; lung cancer (select cases) [70,71,72]	Meningioma: radioresistant histologies, skull-base location, and prior irradiation limiting EBRT. Lung: selected locally advanced or recurrent tumors adjacent to critical structures	Small prospective series and institutional experiences demonstrate technical feasibility, acceptable toxicity, and radiographic responses. High-grade meningioma reported a low local recurrence rate of 22%. These indications remain exploratory, primarily in early-phase or pilot clinical programs.
Cutaneous angiosarcoma; chondrosarcoma [27,79]	Rare, aggressive, and radioresistant tumors; poor control with conventional radiotherapy; frequent superficial or multifocal involvement	Phase I Japanese studies and recent international case reports describe meaningful tumor regression and durable local control in heavily pretreated patients. These indications highlight BNCT’s potential role in orphan or ultra-refractory malignancies.
Recurrent breast cancer (chest wall) [73,74]	Prior radiotherapy precluding further EBRT; superficial but extensive chest-wall disease; need for highly localized retreatment	World’s first exploratory clinical studies recently initiated, evaluating feasibility, toxicity, and imaging-guided boron delivery. This indication is in very early clinical development.

## Data Availability

No new data were created or analyzed in this study. Data sharing is not applicable to this article.

## Abbreviations

ABNS	accelerator-based neutron source
AI	artificial intelligence
BBB	blood–brain barrier
Be	beryllium
BNCT	boron neutron capture therapy
BPA	boronophenylalanine
BSA	beam-shaping assembly
BSH	sodium borocaptate
CT	computed tomography
DC	direct current
DTL	drift-tube linac
EBRT	external-beam radiotherapy
GBM	glioblastoma multiforme
HNSCC	head and neck squamous cell carcinoma
IAEA	International Atomic Energy Agency
LAT1	L-type amino acid transporter 1
LET	linear energy transfer
Li	lithium
linac	linear accelerator
MC	Monte Carlo
ML	machine learning
MRI	magnetic resonance imaging
PET	positron emission tomography
RFQ	radio-frequency quadrupole
RPT	radiopharmaceutical therapy
TBR	tumor-to-blood ratio
TNR	tumor-to-normal tissue ratio
